# Large mesenteric hematoma after extracorporeal shock wave lithotripsy for pancreatic stones: A case report

**DOI:** 10.1097/MD.0000000000013114

**Published:** 2018-11-02

**Authors:** Yu Liu, Lu Hao, Li-Sheng Wang, Teng Wang, Zhao-Shen Li, Liang-Hao Hu, Zheng-Lei Xu

**Affiliations:** a Department of Gastroenterology, Gongli Hospital; b Department of Gastroenterology, Changhai Hospital, The Second Military Medical University, Shanghai; c Department of Gastroenterology, Hainan Branch of Chinese PLA General Hospital, Sanya; d Digestive Endoscopy Center, Changhai Hospital, The Second Military Medical University, Shanghai; e Department of Gastroenterology, The Second Clinical Medical College (Shenzhen People's Hospital), Jinan University, Guangdong, China.

**Keywords:** complication, extracorporeal shock wave lithotripsy, mesenteric hematoma, pancreatic stones

## Abstract

**Rationale:**

Mesenteric hematoma after extracorporeal shock wave lithotripsy (ESWL) for pancreatic stones is a very rare complication which has never been reported before.

**Presenting concerns:**

We reported a case of a 36-year-old male diagnosed as chronic pancreatitis with pancreatic stones and a large pancreatic pseudocyst. He underwent 3 repeated sessions of pancreatic ESWL (P-ESWL). After the last session of P-ESWL, he complained of dizziness. Physical examination revealed a large mass in the right abdomen.

**Diagnosis:**

Emergent upper abdominal computerized tomography (CT) revealed this mass is a mesenteric hematoma with the size of 8.2 cm × 11.7 cm in the right abdominal cavity after P-ESWL and there was no sign of intestinal obstruction.

**Interventions:**

With close monitoring of vital signs, the patient received conservative treatment for several days. Dynamic abdominal ultrasound monitoring revealed the mesenteric hematoma had organized.

**Outcomes:**

Vital signs of the patient were stable after fluid transfusion. Three-month follow-up CT showed the mesenteric hematoma had absorbed completely.

**Lessons:**

Mesenteric hematoma rarely occurs after P-ESWL and it alerts us the importance of considering uncommon complications after P-ESWL. If mesenteric hematoma occurs after P-ESWL, conservative treatment could be the first choice while surgery can also be considered.

## 1 Introduction

Pancreatic stone is a sequel of chronic pancreatitis (CP) and may obstruct the pancreatic ducts and produce ductal hypertension, which leads to pain, the cardinal feature of CP.^[1]^ Extracorporeal shock wave lithotripsy (ESWL) has been recommended for the management of large pancreatic stones since 1987.^[2]^ It is proved to be a less invasive and lower-morbidity procedure compared with surgery in the past 30 years.^[3–6]^ However, despite it is a minimal-invasive therapy, it does have complications. The major complications of pancreatic ESWL (P-ESWL) include post-ESWL pancreatitis, bleeding, infection, steinstrasse, and perforation.^[7]^ Mesenteric hematoma is a very rare complication which has never been reported before. At our endoscopic center, over 800 sessions of P-ESWL were performed per year. Among 6000 sessions of P-ESWL we had performed, only one patient developed symptomatic large mesenteric hematoma. Here we present the patient developed large mesenteric hematoma after P-ESWL.

## 2 Case report

The study was approved by the Ethics Committee of Changhai Hospital, The Second Military Medical University, Shanghai, China. Written informed consent was obtained from the patient for publication of this report.

**Figure 1:**
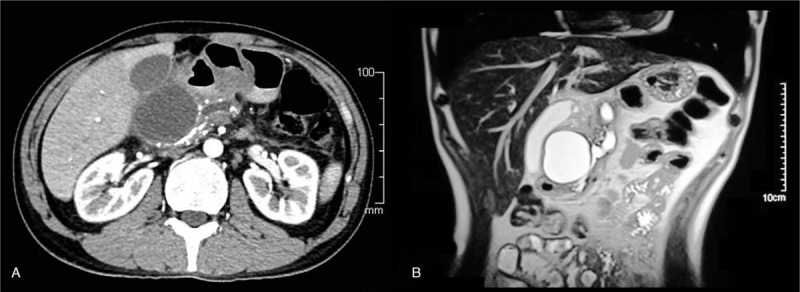
(A) The computerized tomography (CT) scan shows the upstream dilation of the pancreatic with the radiopaque stones in the pancreatic body and a large pancreatic pseudocyst (PPC) with the size of about 5.0 cm × 6.3 cm of the pancreas. (B) Magnetic resonance cholangiopancreatography reveals the upstream dilation of the pancreatic with the radiopaque stones in the pancreatic body and a large pancreatic pseudocyst (PPC) with the size of about 5.0 cm × 6.3 cm of the pancreas. PPC = pancreatic pseudocyst.

A 36-year-old male was admitted to our department because of intermittent upper abdominal pain for five years. Computed tomography (CT) and magnetic resonance cholangiopancreatography revealed the upstream dilation of pancreatic duct with radiopaque stones and a large pancreatic pseudocyst (PPC) with the size of about 5.0 cm × 6.3 cm (Fig. 1A and B). The examinations confirmed the diagnosis of CP.

**Figure 2:**
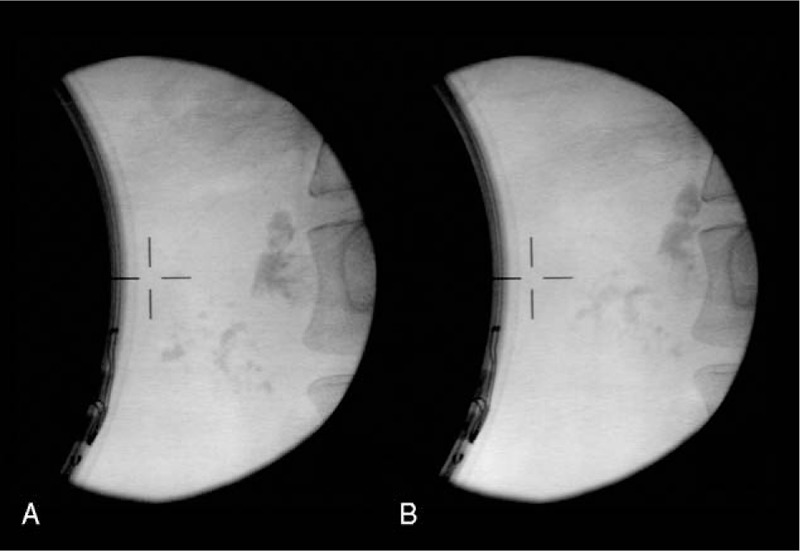
(A) The x-ray image for pancreatic stone of the patient before the first session of extracorporeal shock wave lithotripsy for pancreatic stones (P-ESWL). (B) The x-ray image for pancreatic stone of the patient after 3 sessions of P-ESWL. P-ESWL =  pancreatic extracorporeal shockwave lithotripsy

We have reported the effectiveness and safety of P-ESWL coexisting with PPCs before.^[3]^ Thus, we performed P-ESWL by using a third-generation lithotripter (Delta Compact II, Dornier Med Tech, Wessling, Germany) on the patient in a supine position to pulverize the stones. The patient received combined flurbiprofen and remifentanil via intravenous infusion for analgesia during the procedure. Up to 5000 shock waves were delivered per therapeutic session at an intensity of 6 (16,000 kV) on a scale of 1 to 6 with a frequency of 120 shocks/min. Three repeated sessions of P-ESWL were performed for 3 successive days (Fig. 2A and B). Serum amylase and lipase were all within the normal range after each session of P-ESWL.

**Figure 3:**
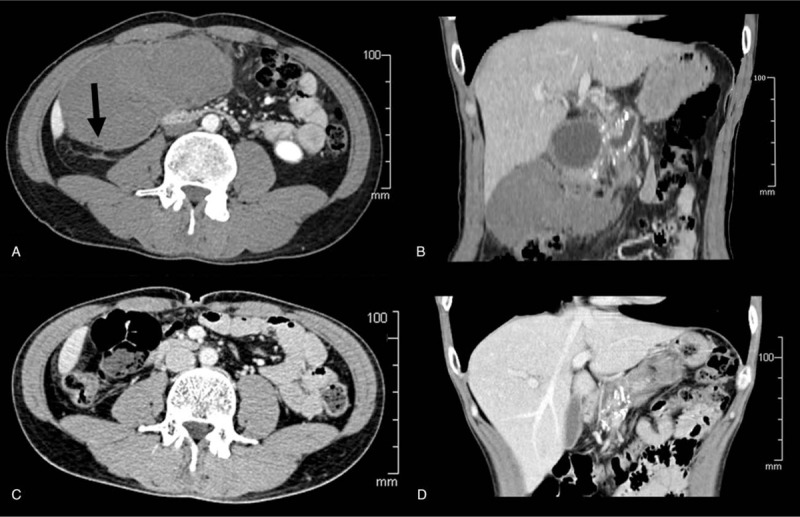
(A) The emergent computerized tomography (CT) scan shows a large mesenteric hematoma with the size of 8.2 cm × 11.7 cm in front of the head of pancreas in the right abdominal cavity. Gas in the hematoma (arrow) verifies the hematoma comes from mesentery. (B) CT scan reveals the large mesenteric hematoma with the size of 8.2 cm × 11.7 cm in front of the head of pancreas in the right abdominal cavity. (C) CT scan shows the large mesenteric hematoma has completely absorbed after 3 months. (D) CT scan reveals the large mesenteric hematoma has completely absorbed, the large pancreatic pseudocyst has disappeared and the pancreatic stones have decreased significantly. CT = computed tomography.

Upon admission, blood coagulation, platelet count and function were within the normal range and no positive findings in physical examination. However, the patient started to complain of dizziness 12 hours after the last therapeutic session of P-ESWL. Physical examination showed a large mass in the right abdomen at the umbilical plane with mild tenderness. The blood tests showed hemoglobin dropped from 137 g/L (upon admission) to 98 g/L. The blood pressure dropped from 120/90 to 110/80 mm Hg. Emergent upper abdominal CT revealed the large mass was a large mesenteric hematoma with the size of 8.2 cm × 11.7 cm which occupied the root of mesentery in front of the head of pancreas in the right abdominal cavity (Fig. 3A and B).There was no characteristic findings of acute pancreatitis or intestinal obstruction on CT scans. Fluids were transfused and vitals monitored. The patient was advised bed rest and was kept nil per oral. The vital signs were stable after fluid transfusion. Eight days later, dynamic monitoring by abdominal ultrasound verified the organized mesenteric hematoma with the size of 13.2 cm × 6.8 cm, which revealed bleeding had ceased. Thus, the patient was discharged from hospital.

The 3-month follow-up CT showed the mesenteric hematoma and the PPC had been absorbed completely and pancreatic stones decreased significantly (Fig. 3C and D). Endoscopic retrograde cholangiopancreatography (ERCP) was performed. The residual pancreatic stones were cleared out with basket. The patient was asymptomatic during the 6 years follow-up.

## 3 Discussion

Chronic pancreatitis is a progressive inflammatory disease characterized with pancreatic stones. Pancreatic stones may cause the obstruction of pancreatic ducts and ductal hypertension, which may lead to abdominal pain and deficiency of pancreatic endocrine and exocrine function.^[8–11]^ Therefore, removing pancreatic stones to drain pancreatic juice is essential. There are many optional treatments to remove pancreatic stones, such as ERCP, P-ESWL, mechanical lithotripsy, laser lithotripsy, surgery and others.^[12–17]^ Among these methods, P-ESWL is a valid and minimally invasive treatment for pancreatic stones for its effectiveness and safety.^[3–6]^ Although P-ESWL is a safe and effective therapeutic modality, complications happen now and then. Complications after P-ESWL including pancreatitis, bleeding, infection, steinstrasse and performation.^[18]^ Acute gastrointestinal mucosal injury after P-ESWL characterized by melena with a high incidence rate of 2.7% is not included in the complications.^[18]^ Bleeding which usually occurs in bounded organ is a rare complication with a low incidence rate of 0.3%.^[18]^ The location of hematoma that has been reported includes: hepatic subcapsular hematoma^[19]^ and hilar hematoma.^[20]^ Mesenteric hematoma is a very rare complication after P-ESWL which has never been reported before.

In our case, the occurrence of the mesenteric hematoma may be caused by 2 major factors as follows: the partial energy release of the shock wave may affect the organs along the conduction pathway and the shock wave generator cannot always locate the pancreatic stones constantly because organs in abdomen moves during respiration.^[21]^ Therefore, this mesenteric hematoma occurrence may be due to the injury of mesenteric vessels during P-ESWL procedure.

As mesenteric hematoma rarely occurs after P-ESWL, experiences of clinical treatment are lacking. Traditionally, mesenteric hematomas could occur after injuries of mesenteric vessels or occur spontaneously. In the first situation, mesenteric hematomas occur after abdominal trauma or abdominal surgery.^[22,23]^ In the second circumstance, mesenteric hematomas that occurs spontaneously are usually associated with coagulogathies, connective tissue disorders, arteriopathy, pancreatitis, or bleeding from visceral artery aneurysms.^[24–28]^ Literatures showed that treatments for mesenteric hematoma remain a controversy. Whether to operate or not on patients with mesenteric hematoma depends on specific condition.^[29]^ In our case, we prepared the surgery team for the patient at the time we found the mesenteric hematoma. However, we preferred to attempt conservative treatment first for the reason that injuries caused by P-ESWL are limited in most time from our experience. We have reported that 634 consecutive patients underwent 1470 ESWL procedures in our hospital between March 2011 and June 2013. In all these procedures, five cases (5/1470, 0.3%) developed bleeding after P-ESWL. Among the 5 cases, one patient with hepatic subcapsular hematoma was treated with percutaneous hematoma drainage and four other patients were under close observation and conservative medical treatment. All bleeding cases were controlled perfectly without surgery.^[18]^ Thus, for this patient, blood tests were carried out and physical examination was performed to estimate the state of the bleeding. Vital signs were monitored closely. Fluid was transfused to stabilize the blood pressure. After fluid transfusion, the patient's vital signs were stable and the symptoms relieved. The dynamic abdominal ultrasound revealed that the mesenteric hematoma had organized 8 days after. The hematoma in our case increased from 8.2 to 13.2 cm in one dimension after 8 days. The reason is as following. Once the mesenteric hematoma forms, inflammatory response starts. Inflammatory cells like lymphocytes, plasma cells, granulocytes, and phagocytes are activated, accumulated and infiltrated in the hematoma.^[30,31]^ The exudation of fibrin and inflammatory cells makes the hematoma larger on CT scan.^[32,33]^ Therefore, the mesenteric hematoma grows larger after eight days. Three-month follow-up CT showed the mesenteric hematoma was absorbed spontaneously. Hence, conservative treatment was proved to be effective and surgery was not needed.

Despite we handled the rare complication after P-ESWL successfully, there are some limitations in approach to this case. Firstly, to choose conservative treatment for such patients without any experience may be risky although we have monitored the vital signs closely. Secondly, the guiding role of this case for future conditions may be limited as the situation of such complications after P-ESWL is unpredictable. Therefore, the treatment for such patients still needs to be explored in the future.

## 4 Conclusion

In conclusion, this case report is the first to describe a large mesenteric hematoma after P-ESWL. Mesenteric hematoma rarely occurs after P-ESWL and it alerts us the importance to consider uncommon complications after P-ESWL. If mesenteric hematoma occurs after P-ESWL, conservative treatment could be the first choice while surgery should be prepared as well. For conservative treatment, monitoring vital signs and blood loss, and estimating the state of mesenteric hematoma is vital for keeping the patient stable.

## Author contributions

**Conceptualization:** Zhao-Shen Li.

**Formal analysis:** Liang-Hao Hu.

**Funding acquisition**: Liang-Hao Hu, Zheng-Lei Xu.

**Investigation**: Liang-Hao Hu.

**Project administration**: Yu Liu, Lu Hao, Teng Wang.

**Writing – original draft**: Yu Liu, Lu Hao, Teng Wang.

**Writing – review & editing**: Li-Sheng Wang, Liang-Hao Hu, Zhao-Shen Li.
